# Relationship between tectonic tremors and 3-D distributions of thermal structure and dehydration in the Alaska subduction zone

**DOI:** 10.1038/s41598-022-10113-2

**Published:** 2022-04-14

**Authors:** Kaya Iwamoto, Nobuaki Suenaga, Shoichi Yoshioka

**Affiliations:** 1 Department of Planetology, Graduate School of Science, Kobe University, Rokkodai-cho 1-1, Nada ward, Kobe, 657-8501 Japan; 2 Research Center for Urban Safety and Security, Kobe University, Rokkodai-cho 1-1, Nada ward, Kobe, 657-8501 Japan

**Keywords:** Tectonics, Solid Earth sciences, Seismology

## Abstract

The Alaska subduction zone is characterized by a subducting oceanic plateau, which is referred to as the Yakutat terrane. Tectonic tremors occur in this zone, and there are few volcanoes above the subducted Yakutat terrane. In this study, we performed a 3-D numerical simulation of a thermal structure associated with the simultaneous subduction of the Yakutat terrane and Pacific plate to elucidate the mechanism of tectonic tremors, which typically involve the presence of water. We calculated the water content distribution near the slab surface by using the thermal structure obtained from our simulation and phase diagrams of the hydrous minerals included in the slab. As a result, dehydration from the marine sedimentary layer and oceanic crust was observed near the area where tectonic tremors occurred. Tectonic tremors occur only in the Yakutat terrane because the marine sedimentary layer and oceanic crust are thicker there, and the total amount of water content in these layers is higher; therefore, the amount of dehydration is also higher there than in the Pacific plate. Additionally, there are few volcanoes above the subducted Yakutat terrane because little water remains within the slab beneath the volcanic chain where magma is produced.

## Introduction

**Figure 1:**
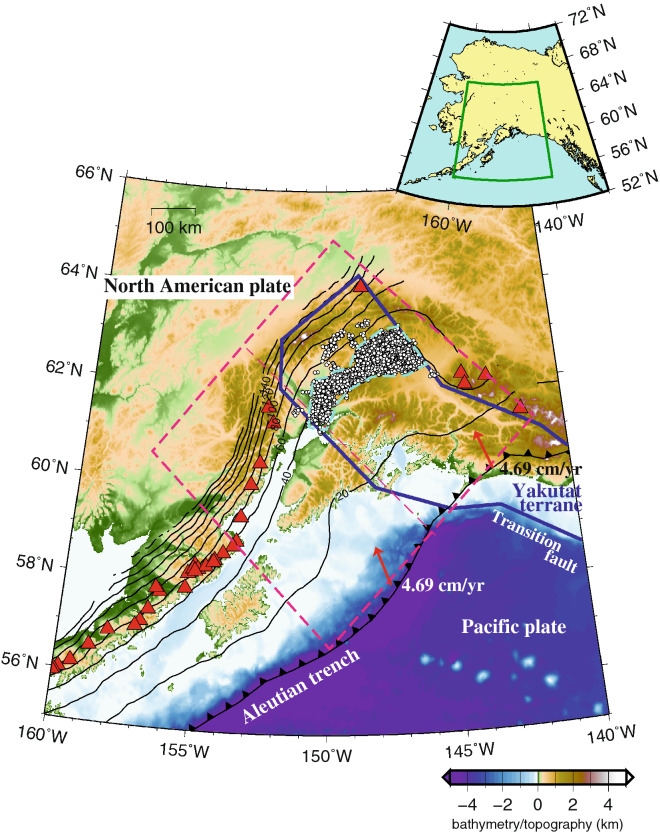
Tectonic map of the Alaska subduction zone. The tectonic map shows the area within the solid green box in the inset. The thick blue solid line represents the Yakutat terrane^1^. The white circle indicates the epicentre^6^ of tectonic tremors, and the light blue dashed line indicates the tectonic tremor occurrence area used in Figs. 2, 3, 4, and 5. The thick barbed black line indicates the Aleutian Trench, the thin black lines indicate the isodepth contours of the upper surface of the subducted oceanic plate by Slab 2^26^ with a contour interval of 20 km, and the red triangles indicate volcanoes. Red arrows represent the current (0 Ma) subduction direction of the oceanic plate at the trench calculated from Matthews et al.^27^. The pink dashed box is the model region in this study, and the pink dashed line in the centre dividing the model region into the northeast and southwest sides represents the boundary between the subducted Yakutat terrane and the subducted Pacific plate in our model. The map was created by using the Generic Mapping Tools (GMT)^25^ (version: GMT 4.5.7, URL link: https://www.generic-mapping-tools.org/download/).

In the Alaska subduction zone, the Pacific plate is being subducted beneath the North American plate along the Aleutian Trench in a north-northwest direction with a convergence rate of approximately 4.7 cm/year (Fig. 1). Recent studies have shown that the Yakutat terrane is being subducted at its eastern end^1^. The seismic velocity structure of the Yakutat terrane indicates that it consists of an oceanic plateau with a 4.5- to 7.5-km-thick marine sedimentary layer and a 24- to 27-km-thick oceanic crust^2^. In the Alaska subduction zone, megathrust earthquakes such as the 1964 Alaska earthquake (Mw 9.1) occurred near the plate boundary, and slow earthquakes, such as long-term slow slip events (L-SSEs) and tectonic tremors, occurred on the downdip side of the seismogenic zone. L-SSEs occurred at the plate interface of the subducted Yakutat terrane in 1998–2001 and 2009–2013^3,4^ and at the upper surface of the Pacific slab in 2010–2012^5^. Tectonic tremors occur only on the side of the subducted Yakutat terrane. Wech^6^ examined 11,356 tectonic tremors that occurred from 2007 to 2015 and determined their epicentres. Additionally, few volcanoes are present above the subducted Yakutat terrane.

Qu et al.^7^ obtained a 3-D thermal structure by using numerical modelling for the region from the Aleutian Islands to south-central Alaska, which is located southwest of our model domain. They discussed “wedge earthquakes”, which occur in the accretionary wedges that are located near the trench and cold mantle wedges, and discussed the relationships along them, the slab temperature distribution, and dehydration. They argued that the gradual decrease in the slab dip angle from the Aleutian Islands to south-central Alaska was responsible for the differences in the epicentral distributions of wedge earthquakes in the along-arc direction. Based on the epicentral distribution of the wedge earthquakes in this region, they found that the wedge earthquake epicentre that was farthest from the trench was located above the slab surface, where the depth reached 60 km. They concluded that the slab was fully dehydrated at slab surface depths ranging from 60 to 100 km, and water was transported into the mantle wedge and continental crust, which caused the wedge earthquakes.

Iwamoto et al.^8^ constructed a 3-D thermomechanical model that was associated with the simultaneous subduction of the Yakutat terrane and Pacific plate in the Alaska subduction zone. They estimated that the interplate temperatures for the source region of the 1964 Alaska earthquake (Mw 9.1) with coseismic slip amounts greater than 2 m were 150–450 °C and that the interplate temperatures for the slip areas with cumulative slip amounts greater than 5 cm in the northeastern 2009–2013 L-SSEs and in the southwestern 2010–2012 L-SSEs were 325–600 °C and 300–400 °C, respectively.

In this study, we constructed a 3-D thermomechanical model that was similar to that reported by Iwamoto et al.^8^ to determine the water content distribution near the slab surface by using phase diagrams of the hydrous minerals contained in the slab. We discuss the relationships among dehydration and tectonic tremors and volcanic chains in the Alaska subduction zone. In the water content calculations, we newly introduced the marine sedimentary layer and included the thicknesses of the marine sedimentary layer and oceanic crust separately for the Pacific plate and the Yakutat terrane.

## Results

### Temperature distribution

In this numerical simulation, the effective friction coefficient ${\mu }^{{\prime}}$ is the parameter that governs the frictional heating at the plate boundary. We calculated four models with different values of ${\upmu }^{{{\prime}}}$, namely, 0.008500, 0.01275, 0.01700, and 0.02125. We calculated the heat flow values from the temperature distributions of the respective models and selected the most suitable model with the smallest weighted root mean square (RMS) value of the residuals between the observed and calculated heat flows. As a result, the RMS value was smallest when ${\mu }^{{\prime}}=0.01275$ (Table S2). Iwamoto et al.^8^ conducted numerical modelling of a 3-D thermal structure, which differed from this study only in the geometry of the subducted Yakutat terrane. In our model, the depth distribution of the plate interface in the northeastern part is at most approximately 30 km shallower than that in Iwamoto et al.^8^ (Fig. S1a,d). Therefore, when comparing the models with the optimum effective friction coefficient, the temperature distribution of the slab surface in our model is as great as 100–200 °C lower in the region where the slab geometry was modified, especially on its downdip side (Fig. S2d). However, in our model, the temperature ranges of the interplate seismic events are exactly the same as those obtained from Iwamoto et al.^8^ because the area with a modified slab geometry does not overlap with the source regions of the 1964 Alaska earthquake and the L-SSEs. We estimated that the slab surface temperatures of the 1964 Alaska earthquake, northeastern 2009–2013 L-SSEs, and southwestern 2010–2012 L-SSEs are 150–450 °C, 325–600 °C, and 300–400 °C, respectively.

**Figure 2:**
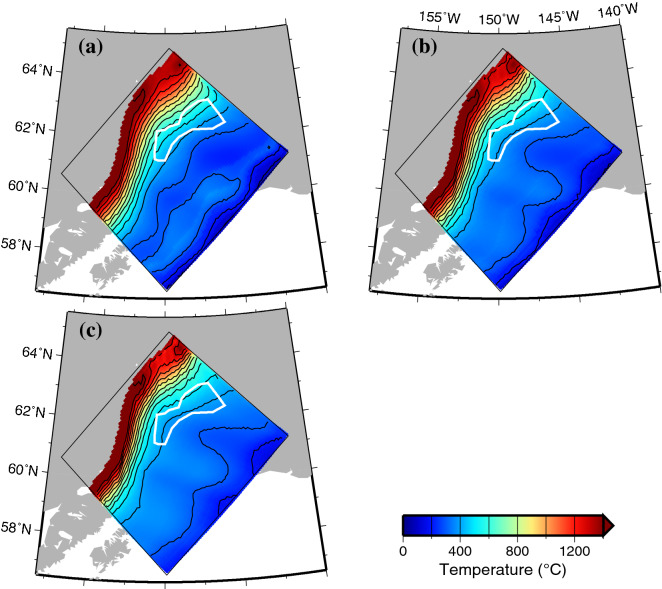
Temperature distribution in the slab at present (0 Ma). The thin black box represents the model region. The temperature distribution is plotted only in the region where the depth of the slab surface is shallower than the bottom of the model (200 km), with a contour interval of 100 °C. The white line indicates the area where tectonic tremors occur, as shown in Fig. 1. (**a**) The slab surface (0 km). (**b**) 6 km depth from the slab surface. (**c**) 10 km depth from the slab surface. The map was created by using the Generic Mapping Tools (GMT)^25^ (version: GMT 4.5.7, URL link: https://www.generic-mapping-tools.org/download/).

Similar to Iwamoto et al.^8^, the temperature distribution at the present time (0 Ma) that was obtained from our numerical simulation, shows that the temperatures increase with the slab surface depth (Fig. 2). We also found that the thermal gradient along the subduction direction is small because the slab geometry is shallow and flat from the trench side to the central part of the model domain. Because the seafloor age of the Yakutat terrane is older than that of the Pacific plate, the surface temperatures of the subducted Yakutat terrane tend to be lower than those of the Pacific plate for the same depth. Additionally, at the same location, the temperatures at the slab surface (0 km) are slightly higher than those at depths of 6 km and 10 km from the slab surface because the slab surface is heated by friction at the boundary with the continental crust on the trench side and the slab surface is exposed to the hot mantle on the inland side.

Although the epicentres of the tectonic tremors in south-central Alaska were determined by Wech^6^, their depths were not determined. Tectonic tremors have been observed near plate interfaces in subduction zones such as southwest Japan, Cascadia, Mexico, southern Chile, and Hikurangi (e.g., Ide^9^). Ide^9^ determined the hypocentres of the tectonic tremors in these subduction zones by analysing their seismic waveforms. They found that tectonic tremors occur near the plate interfaces. Therefore, in this study, we assumed that the tectonic tremors in south-central Alaska take place at the plate interface. We used the temperature and depth at the slab surface that were obtained from our numerical simulation for each tectonic tremor and found that the temperature range at the locations of the tectonic tremors was estimated to be 484 ± 71 (1σ) °C.

### Water content

**Figure 3:**
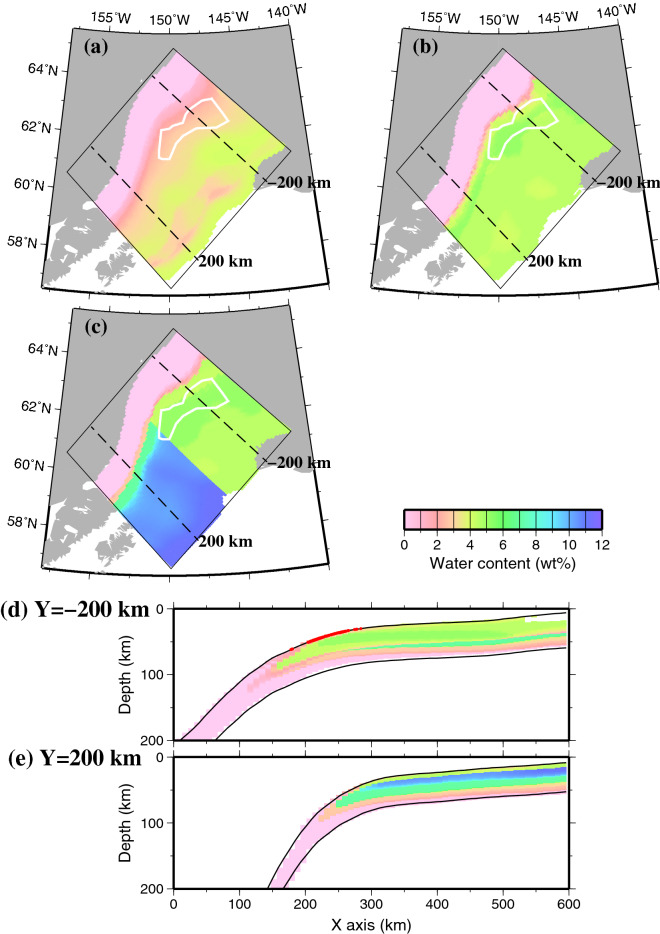
Water content distribution in the slab at present (0 Ma). The thin black box represents the model region. The water content distribution is plotted only in the region where the depth of the slab surface is shallower than the bottom of the model (200 km) and where the temperature is higher than 200 °C, for which values of phase diagram data exist. The white line indicates the area where tectonic tremors occur, as shown in Fig. 1, and the black dashed lines indicate the location of profile Y = − 200 km and Y = 200 km. (**a**) The slab surface (0 km). (**b**) 6 km depth from the slab surface. (**c**) 10 km depth from the slab surface. (**d**) The vertical cross section along profile Y = − 200 km. The water content distribution is plotted only in the region where the temperature is higher than 200 °C, for which values of phase diagram data exist. The two solid black lines represent the top and bottom surfaces of the slab. The red solid circles represent the hypocentres of tectonic tremors within a one-sided width of 1 km. (**e**) Same as (**d**) except the vertical cross section along profile Y = 200 km. The map was created by using the Generic Mapping Tools (GMT)^25^ (version: GMT 4.5.7, URL link: https://www.generic-mapping-tools.org/download/).

Based on the duration and mobility of the tectonic tremors, it is considered that the water that is produced by dehydration from hydrous minerals in the slab is transported to the plate boundary and decreases the effective normal stress, which causes tectonic tremors (e.g., Obara^10^). Therefore, we calculated the water contents of the hydrous minerals in the slab based on the relationship between depth and temperature near the plate boundary that was obtained from our thermal structure model. The Yakutat terrane has a considerably thicker marine sedimentary layer and oceanic crust than those in the Pacific plate. We considered that the differences in the thicknesses of these layers would affect the water content distributions in the Yakutat terrane and Pacific plate. Therefore, we divided the slab into three layers, namely, the marine sedimentary layer, oceanic crust, and slab mantle, and we calculated the water content distribution of each layer (Fig. 3). For the Pacific plate, we set the marine sedimentary layer to be 2 km thick from the slab surface, the oceanic crust to be 5 km thick below that, and the slab mantle was defined to extend below the oceanic crust to the bottom of the slab (Table S3). On the other hand, for the Yakutat terrane, we defined the marine sedimentary layer to be 6 km thick from the slab surface, the oceanic crust to be 25 km thick below that, and the slab mantle was defined to extend below the oceanic crust to the bottom of the slab^2^. To calculate the water contents, we used the turbidite phase diagram^11^ for the marine sedimentary layer, MORB phase diagram^12^ for the oceanic crust, and ultramafic rock phase diagram^12^ for the slab mantle (Fig. S3).

At the surface of the subducted Yakutat terrane, the variations in water contents in the subduction direction (− x) show that the water contents gradually change near the area where tectonic tremors occur (Fig. 3a). These spatial changes are attributed to the dehydration that is associated with the phase transformation from phengite lawsonite blueschist to amphibole phengite zoisite eclogite in the turbidites of the marine sedimentary layer (Fig. S3a). At a depth of 6 km from the slab surface, the water contents change significantly near the downdip side of the tectonic tremor-generating area (Fig. 3b). This spatial change is due to the dehydration that is associated with the phase transformation from lawsonite blueschist to lawsonite eclogite in the MORB of the oceanic crust (Fig. S3b). At a depth of 10 km from the slab surface, dehydration from the MORB is dominant in the Yakutat terrane, which is similar to the distribution at a depth of 6 km. On the other hand, the Pacific plate consists of a slab mantle at a depth of 10 km, and the ultramafic rock transforms from brucite to antigorite, chlorite, and amphibole in sequence in association with subduction (Figs. 3c and S3c). The p–T paths at the slab surface (0 km) show that the temperatures increase with depth, then decrease, and then increase again (Fig. S3a). This is due to the frictional heating at the boundary between the continental upper crust and slab surface, which causes a rapid increase in temperature at shallow depths. At greater depths, the temperatures decrease because frictional heating does not occur at the boundary with the continental lower crust and mantle, which are convective regions in our numerical simulation, and the temperatures then increase again due to the hot mantle at deeper depths.

The vertical cross section in the subduction direction also shows a significant change in the water contents near the downdip side of the tectonic tremors (Fig. 3d). As described above, this is due to the phase transformation from phengite lawsonite blueschist to amphibole phengite zoisite eclogite in the turbidites of the marine sedimentary layer from the slab surface (0 km) to a depth of 6 km and to the phase transition from lawsonite blueschist to lawsonite eclogite in the MORB of the oceanic crust in a depth range of 6–31 km from the slab surface. On the other hand, the Pacific plate has a thicker slab mantle, and the decreased water contents of the ultramafic rocks are dominant (Fig. 3e).

### Dehydration gradient

**Figure 4:**
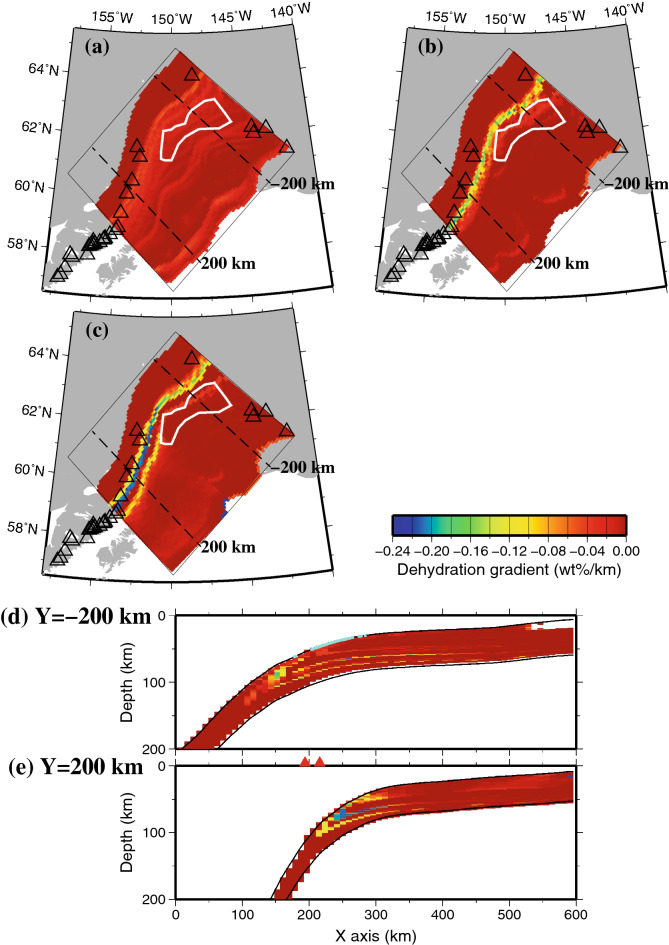
Dehydration gradient distribution in the slab at present (0 Ma). The thin black box represents the model region. The dehydration gradient distribution is plotted only in the region where the depth of the slab surface is shallower than the bottom of the model (200 km) and where the temperature is higher than 200 °C, for which values of phase diagram data exist. The white line indicates the area where tectonic tremors occur, as shown in Fig. 1, and the black dashed line indicates the location of profile Y = − 200 km and Y = 200 km. The open triangles represent volcanoes. (**a**) The slab surface (0 km). (**b**) 6 km depth from the slab surface. (**c**) 10 km depth from the slab surface. (**d**)The vertical cross section along profile Y = − 200 km. The dehydration gradient distribution is plotted only in the region where the temperature is higher than 200 °C, for which values of phase diagram data exist. The two solid black lines represent the top and bottom surfaces of the slab. The light blue solid circles represent the hypocentres of tectonic tremors within a one-sided width of 1 km. (**e**) The vertical cross section along profile Y = 200 km. The red solid triangles represent volcanoes within a one-sided width of 50 km. The others are the same as (**d**). The map was created by using the Generic Mapping Tools (GMT)^25^ (version: GMT 4.5.7, URL link: https://www.generic-mapping-tools.org/download/).

From the water content distribution, we calculated the differences in water content per unit length in the subduction direction, which are referred to as the dehydration gradient^13^ (Fig. 4). The larger the absolute value of the dehydration gradient is, the larger the decrease in the water content. This suggests that the dehydration from hydrous minerals is significant in such a region. In the marine sedimentary layer, the spatial distribution of the dehydration gradient shows that dehydration is gradually identified from the updip side (~ 0.02 wt%/km) of the tectonic tremor-occurring area and that the dehydration gradient is the largest on the downdip side (~ 0.04 wt%/km) (Fig. 4a). In the oceanic crust, there is no dehydration on the updip side of the tectonic tremor-occurring area, and the dehydration gradient is large in a narrow area on the downdip side (Fig. 4b). The reason for this difference in the distribution of the dehydration gradients means that the water contents of the turbidites in the marine sedimentary layer change gradually, while the water contents in the MORB in the oceanic crust change rapidly due to the dehydration that is associated with the phase transformations (Fig. S3a,b). In the slab mantle, three bands of dehydration were identified (Fig. 4c). The shallowest band corresponds to the phase transition from brucite to antigorite, with a dehydration gradient of approximately 0.10–0.12 wt%/km. The middle band represents the phase transition from antigorite to chlorite, with a gradient of approximately 0.22 wt%/km, which is the largest dehydration gradient of the three. The deepest band corresponds to the phase transition from chlorite to amphibole, with a dehydration gradient of approximately 0.14 wt%/km. The vertical cross section in the x–z plane also shows that the dehydration gradients increase near the downdip side of the tectonic tremor-generating area (Fig. 4d). Thus, dehydration occurs not only on the slab surface but also inside the slab, and the water inside the slab may be transported to the plate interface.

**Figure 5:**
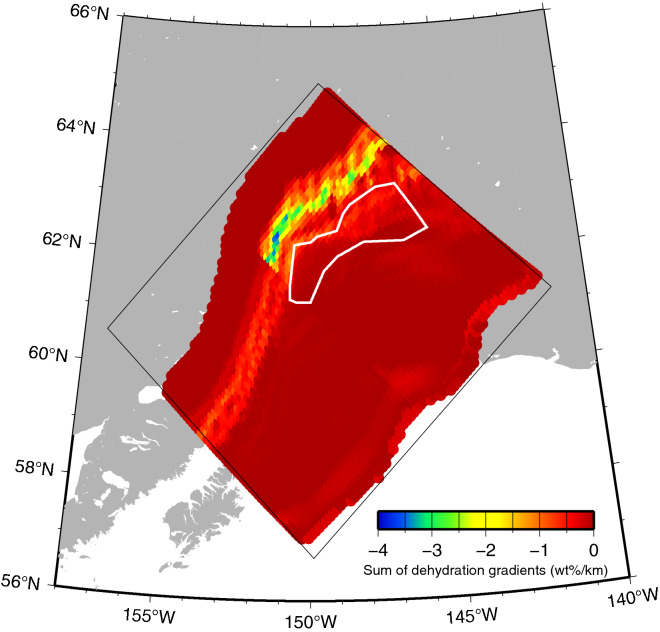
The vertical sum of the dehydration gradients from the slab surface to the slab Moho. The thin black box represents the model region. The vertical sum is plotted only in the region where the depth of the slab surface is shallower than the bottom of the model (200 km) and where the temperature is higher than 200 °C, for which values of phase diagram data exist. The white line indicates the area where tectonic tremors occur, as shown in Fig. 1. The map was created by using the Generic Mapping Tools (GMT)^25^ (version: GMT 4.5.7, URL link: https://www.generic-mapping-tools.org/download/).

In this study, we calculated the vertical sum of the dehydration gradients from the slab surface to the slab Moho. In other words, we calculated the vertical sum of the dehydration gradients from the slab surface (0 km) to a depth of 7 km below for the Pacific slab and that from the slab surface (0 km) to a depth of 31 km for the subducted Yakutat terrane. As a result, the vertical sum of the dehydration gradients on the downdip side of the tectonic tremor area was the largest and was remarkable (Fig. 5). This is largely because the Yakutat terrane has a thick oceanic crust of 25 km. Therefore, the distribution of the total dehydration gradient in the Yakutat terrane (Fig. 5) is similar to the distribution of the dehydration gradient of the oceanic crust (Fig. 4b).

Incidentally, we also calculated the sum of the dehydration gradients from the slab surface to the slab Moho when the thickness of the sedimentary layer in the Yakutat terrane is assumed to be 2 km, whose thickness is the same as that of the Pacific plate. In this calculation, we did not change the other thicknesses: the oceanic crust in the Yakutat terrane is 25 km thick, and the oceanic sedimentary layer and oceanic plate in the Pacific plate are 2 km and 5 km thick, respectively. In this case, there were no significant differences from the distribution of the summed dehydration gradients shown in Fig. 5, which were large near the tectonic tremor-generating area (Fig. S4). This is because the dehydration from the MORB of the oceanic crust is more dominant in the sum of the dehydration gradients than that from the turbidites of the sedimentary layer.

## Discussion

As mentioned above, the temperature range at the slab surface in the region where tectonic tremors occur was found to be 413–555 °C (± 1σ) in our numerical simulation. Additionally, the turbidites in the marine sedimentary layer transform from the phengite lawsonite blueschist phase to the amphibole phengite zoisite eclogite phase, and the MORB in the oceanic crust transforms from the lawsonite blueschist phase to the lawsonite eclogite phase near the tectonic tremor-generating area (Fig. 3).

Suenaga et al.^14^ constructed a 2-D thermal structure model to estimate the temperature range for the tectonic tremors that occurred in the Kyushu district, southwest Japan. Tectonic tremors have been observed in only a limited area near Miyazaki Prefecture, which is located in the southeastern part of Kyushu and is located within the mantle wedge at depths of approximately 30–40 km, according to the JMA unified earthquake catalogue. They estimated that the temperature range of the area with tectonic tremors was 450–650 °C. They showed that the MORB in the oceanic crust of the slab transforms from the lawsonite blueschist phase to the lawsonite eclogite phase beneath the area of tectonic tremors, which is consistent with our study. Suenaga et al.^15^ calculated the dehydration gradient from a 3-D thermal structure model in Hikurangi. They found that the dehydration gradient is 0.2 wt%/km for the MORB of the slab in the tectonic tremor-generating area. In our study, the dehydration gradients are approximately 0.04 wt%/km in the sedimentary layer at the slab surface and approximately 0.12 wt%/km in the MORB at a depth of 6 km from the slab surface in the Yakutat terrane (Fig. 4a,b). The order of the dehydration gradient of the MORB obtained from this study is the same as that presented in Suenaga et al.^15^.

Qu et al.^7^ determined the water content distribution of the slab in the Alaska subduction zone. They concluded that the slab is completely dehydrated in the 60–100 km depth range along the slab surface and that the released water was transported to the mantle wedge. The water content distribution obtained from our study shows that the slab is completely dehydrated in the depth range of approximately 70–110 km along the slab surface, and from this area to the bottom of the model, the water contents in the slab are approximately 0 wt% (Fig. 3d). The depth at which dehydration was complete was slightly deeper than that reported by Qu et al.^7^. This is because the MORB phase diagram was used to calculate the water content near the slab surface in Qu et al.^7^, whereas we newly introduced the marine sedimentary layer in the top of the slab and used the turbidite phase diagram, which allowed the hydrous minerals to remain at greater depths than indicated by the MORB phase diagram. In addition, the introduction of the Yakutat terrane, which is older than the Pacific plate in seafloor age, may cause an overall decrease in the temperature distribution of the subducted Yakutat terrane and transport hydrous minerals to greater depths.

In general, the dehydration that is associated with the phase transformations of hydrous minerals in slabs occurs in regions with large dehydration gradients. The dehydrated water is transported to the plate boundary, decreases the effective normal stress there, and causes tectonic tremors. In our study, both the marine sedimentary layer and oceanic crust have large dehydration gradients near the area where tectonic tremors occur (Fig. 4a,b). Accordingly, the water that is produced by dehydration from the slab is considered to be responsible for the occurrence of the tectonic tremors in the Alaska subduction zone. Tectonic tremors occur only in the Yakutat terrane in the Alaska subduction zone because the Yakutat terrane has a thick marine sedimentary layer (6 km) and thick oceanic crust (25 km), and the total water contents in these layers are high. Therefore, the amounts of dehydrated water from these layers are also high, which may contribute to tectonic tremors (Fig. 5).

In the Yakutat terrane, which is being subducted at a low dip angle, tectonic tremors occur, and there are few volcanoes. On the other hand, in the Pacific plate, which is being subducted at a high dip angle, tectonic tremors do not occur, but a volcanic chain is present. In southwestern Japan, there are similar spatial changes in the features along the Nankai Trough^12^. In southwestern Japan, tectonic tremors and deep low-frequency earthquakes (LFEs) occur beneath the Shikoku district, which is located to the east of the Kyushu–Palau Ridge, but there are only two volcanoes in the Chugoku district. In contrast, the Kyushu district, which is located to the west of these regions, shows little activity of tectonic tremors and LFEs, while there are 13 active volcanoes. Tatsumi et al.^12^ calculated the water content distribution associated with the subduction of the Philippine Sea plate by using 2-D thermal structure modelling. They concluded that the differences in such characteristics between the Shikoku-Chugoku district and Kyushu district are due to the differences in the dehydration distribution near the plate boundary. In other words, in the Shikoku-Chugoku district, where the oceanic plate is younger and subducts at a lower dip angle, water is released at shallow depths beneath the forearc, which causes tectonic tremors and LFEs. In contrast, in the Kyushu district, where the oceanic plate is older and subducts at a higher dip angle, hydrous minerals are transported to greater depths without dehydration, release water at a depth of approximately 100 km, and generate magma and form volcanoes above.

In the Yakutat terrane, the dehydration in the marine sedimentary layer occurs from the updip side to the downdip side of the tectonic tremor-generating area with a dehydration gradient of approximately 0.04 wt%/km, which is smaller than the gradients in the oceanic crust and slab mantle (Fig. 4a). Additionally, the dehydration in the 25-km thick oceanic crust occurs on the downdip side with a dehydration gradient of approximately 0.12 wt%/km (Fig. 4b). We conclude that the dehydration from these marine sedimentary layers and oceanic crust causes tectonic tremors and that there are few volcanoes because little water remains in the subducted Yakutat terrane beneath them, where magma should be produced. Additionally, the slab mantle in the Yakutat terrane is thinner than that of the Pacific plate and is covered by a thick sedimentary layer and oceanic crust with a combined thickness of 31 km. Therefore, the effect of dehydration from the slab mantle is considered to be limited in the subducted Yakutat terrane.

On the other hand, in the Pacific slab, the sedimentary layer and oceanic crust are much thinner than those of the Yakutat terrane, and dehydration from the slab mantle is considered to play an important role. At depths of 80–100 km beneath the along-arc volcanic chain, the ultramafic rock in the slab mantle transforms from the antigorite phase to chlorite phase with a dehydration gradient of approximately 0.22 wt%/km and then transforms to the amphibole phase with a dehydration gradient of approximately 0.14 wt%/km (Fig. 4c,e). We suggest that this massive amount of dehydration produced the magma that formed the volcanic arc above.

## Conclusions

In this study, we constructed a 3-D thermomechanical model for the Alaska subduction zone, where both the Yakutat terrane and Pacific plate are being subducted. From the temperature distribution near the slab surface that was obtained from our numerical simulation, we calculated the water contents and dehydration gradients and compared them with the area where tectonic tremors occur. The significant results obtained in this study are summarized as follows:

1. The temperature at the slab surface in the tectonic tremor-generating area was found to be 484 ± 71 (1σ) °C.
2. We found that, near the downdip part of the tectonic tremor-generating area, the turbidites in the marine sedimentary layer transform from the phengite lawsonite blueschist phase to the amphibole phengite zoisite eclogite phase, and the MORB in the oceanic crust transforms from the lawsonite blueschist phase to the lawsonite eclogite phase, which result in significant changes in the water contents of the slab. The dehydrated water may be transported to the plate boundary and cause tectonic tremors.
3. The subducted Yakutat terrane has a thick marine sedimentary layer and thick oceanic crust, and the total water contents in these layers are large. As a result, considerable amounts of water are released near the tectonic tremor-generating area, where the water contents varies greatly. Thus, tectonic tremors do not occur in the Pacific slab where these layers are thin, whereas they occur only in the subducted Yakutat terrane.
4. Few volcanoes are located above the subducted Yakutat terrane because little water remains beneath the volcanoes where magma should be produced. The massive amount of dehydration from the mantle slab may produce the magma that forms the volcanic arc above the Pacific slab.

## Methods

### Governing equations

In this study, as in Iwamoto et al.^8^, a 3-D parallelepiped thermomechanical subduction model was constructed. The governing equations describe the conservation of mass, momentum, and energy. We solved these governing equations as a time-marching problem of temperature, mantle flow velocity, and pressure as the unknown parameters by using the finite-difference method. In the energy equation, we considered six terms: advection, thermal conduction, viscous dissipation, adiabatic compression, radioactive heating, and frictional heating at the plate boundary. The viscosity was defined in the form of a composite equation by calculating the viscosities of dislocation creep and diffusion creep^16^.

Frictional heating at the plate boundary is defined as a function of the convergence rate and shear stress acting at the plate boundary^17^. The shear stress in the shallow brittle region ${\tau }_{b}$ is determined by the normal stress ${\sigma }_{n}$ and pore fluid pressure ratio $\lambda$^18^ (Eq. (1)).

1 $${\tau }_{b}=0.85{\sigma }_{n}\left(1-\lambda \right)$$

In this numerical simulation, we calculated models with different values of the pore fluid pressure ratio λ, namely, 0.975, 0.980, 0.985, and 0.990. The relationship between the effective friction coefficient ${\upmu }^{{{\prime}}}(={\tau }_{b}/{\sigma }_{n})$ and pore fluid pressure ratio $\lambda$ is provided by Eq. (2).

2 $${\mu }^{{\prime}}=0.85\left(1-\lambda \right)$$

Thus, for the above-described values of λ, the corresponding effective friction coefficients ${\upmu }^{{{\prime}}}$ are 0.02125, 0.01700, 0.01275, and 0.008500, respectively. Frictional heating at the plate boundary is assumed in the upper crust (0–16 km depth), which is the thermally conductive layer. On the other hand, the shear stress in the deep ductile region is defined to be a function of the shear strain rate and temperature^19^. Smaller values of the two shear stresses were adopted.

### Evaluation of the thermal models by using heat flow data

To evaluate the validity of the thermal structure that was obtained from our numerical simulation, we calculated the weighted RMS values of the residuals between the observed heat flow^20,21^ and the heat flow calculated from our models and selected the optimum model with the smallest RMS value, which was calculated from Eq. (3).

3 $$RMS=\sqrt{\frac{1}{n}\sum_{i=1}^{n}\frac{{d}_{i}}{{d}_{ave}}{(obs}_{i}{-{cal}_{i})}^{2}}$$

where $n$ is the number of stations, ${d}_{i}$ is the distance to the nearest adjacent station, ${d}_{ave}$ is the average value of ${d}_{i}$, ${obs}_{i}$ is the observed heat flow, and ${cal}_{i}$ is the heat flow calculated from our numerical simulation. The RMS values are summarized in Table S2.

## Supplementary Information

Supplementary Information.

## Data Availability

We use Batir et al.^20^ and the Global Heart Flow Database^21^ (https://engineering.und.edu/research/global-heat-flow-database/data.htm) for observed heat flow data. The figures were created using the Generic Mapping Tools (https://www.generic-mapping-tools.org/download/) by Wessel and Smith^25^ and the Paraview software (https://www.paraview.org/download/) by Kitware Inc.
