# Supplementary material for: Relationship between tectonic tremors and 3-D distributions of thermal structure and dehydration in the Alaska subduction zone
Source: Sci Rep. 2022 Apr 14;12:6234. doi: 10.1038/s41598-022-10113-2 (PMC9010436; doi:10.1038/s41598-022-10113-2)
Supplement: Supplementary file 1 — Supplementary Information. [file 41598_2022_10113_MOESM1_ESM.pdf]

Supplementary Information for

Relationship between tectonic tremors and 3-D distributions of thermal structure and dehydration in the Alaska subduction zone

Kaya Iwamoto[1,*], Nobuaki Suenaga[2] and Shoichi Yoshioka[2,1]

[1] Department of Planetology, Graduate School of Science, Kobe University, Rokkodai-cho 1-1, Nada ward, Kobe 657-8501, Japan

[2] Research Center for Urban Safety and Security, Kobe University, Rokkodai-cho 1-1, Nada ward, Kobe 657-8501, Japan

*corresponding author

e-mail: k_iwamoto@stu.kobe-u.ac.jp

Contents

Text S1 and S2
Table S1-S3
Figures and figure captions S1-S9
References

Text S1

Model

Fig. S5 shows the 3-D parallelepiped model domain. The +x axis is taken to be perpendicular to the trench axis in the direction from inland to the trench, the +y axis is oriented along the trench axis from the Yakutat terrane side to the Pacific plate side, and the +z axis is vertical in a downwards direction. The model size is $600\ \text{km} \times 700\ \text{km} \times 200\ \text{km}\ (\text{X} \times \text{Y} \times \text{Z})$, and the number of grids is $70 \times 70 \times 60\ (\text{X} \times \text{Y} \times \text{Z})$. The model domain initially consists of the upper crust with depths from 0 to 16 km, lower crust with depths from 16 to 32 km, and mantle with depths from 32 to 200 km. The upper crust is assumed to be a thermally conductive layer, while the lower crust and mantle are assumed to be convective regions. Different values for both the density and thermal conductivity are used for the thermally conductive layer and convective region. In the thermally conductive layer, the density and thermal conductivity are kept constant at 2600 kg/m^3 and 2.5 W/m·K, respectively. On the other hand, the values in the convective region are defined as functions of temperature and depth. Additionally, in the numerical simulation, a cold nose is not defined in the mantle wedge.

The initial temperature distribution is assumed to be represented by a half-space cooling model[S1] with no flow. The boundary conditions for mantle flow are assumed to consist of no flow at the model surface (-z) and permeable at the five other boundaries (Fig. S5). The boundary condition for temperature is defined as a function of the depth and age of the oceanic plate at the trench in the vertical plane (+x), where the subduction of the oceanic plate begins[S2]. The temperature of the model surface (-z) is held constant at 0°C, and the four other boundaries are set to be adiabatic.

The geometry of the upper surface of the subducting plate was created by using the Slab2 model[S3]. However, the results of the seismic velocity structure survey[S4] showed that the depth of the plate surface was approximately 2 km shallower near the trench than that of the Slab2 model, so we set the slab geometry as such. Furthermore, in general, the iso-depth contours of the plate interface are aligned approximately parallel to the trench axis for most of the area but are perpendicular to the trench axis in the northeastern part of the model domain (Fig. S1(a)). The locations of the hypocentres were used to create the slab geometry of Slab2. However, the number of earthquakes is small in the northeastern part of the model domain, so the reliability of the slab geometry may not be high (Fig. S1(b)). Therefore, we changed the slab geometry in this region so that the iso-depth contours were closer to the direction of the trench axis (Text S2 and Fig. S1(d)). The thickness of the subducting plate was defined as a function of the age of the oceanic plate at the trench[S5]. Using these criteria, we fixed the geometry of the top and bottom

surfaces of the subducting oceanic plate and used this as a prescribed guide. We achieved the subduction of the oceanic plate by gradually pouring oceanic plate material into the guide at the convergence rates[S6]. The convergence rates were determined by considering the subduction history based on the past plate rotation model[S7] (Table S1).

The simulation period was set to 18 Myr, which begins from the start of plate subduction in the model domain to the present when the model domain, including the subducting plate, mantle wedge, and continental plate, reaches a nearly steady thermal state. To constrain the obtained thermal structure at the present (0 Ma), we used the observed heat flow data[S8, S9] and constructed a model in which the residuals between the observations and calculations can be minimized (Figure S6).

Text S2

Effect of the subducted Yakutat terrane geometry on the results

The slab geometry used in Iwamoto et al. (2022)[S10] was created based on the Slab2 model[S3]. In the northeastern part of the model domain, the isodepth contours of the upper surface of the slab are aligned almost perpendicular to the trench axis, and there is little depth variation in the subducting direction of the oceanic plate (Fig. S1(a)). When comparing the temperature distribution near the slab surface by using the slab geometry model in Iwamoto et al. (2022)[S10] with that obtained in this study (Fig. 2), for the same effective friction coefficient, the temperature differences were large in the area where the geometry of the subducted Yakutat terrane was modified, especially on its downdip side with a maximum temperature difference of approximately 100-300 °C (Fig. S2(d), (e) and (f)). The water content distribution in the marine sedimentary layer is hardly affected by this difference in temperature distribution, but the MORB in the oceanic crust undergoes a different phase transformation near the northeastern edge of the model from Fig. 3(b) (Fig. S7). For the slab geometry of Iwamoto et al. (2022)[S10], the MORB in the oceanic crust transforms from the lawsonite blueschist phase to the lawsonite eclogite phase at a depth of approximately 70 km for most of the model domain, which is the same as in Fig. 3(b) (Fig. S7(b)). However, near the northeastern edge of the model domain, the MORB in the oceanic crust transforms from the blueschist phase to the amphibole eclogite phase at a depth of approximately 40 km (Fig. S7(b)). Therefore, the maximum dehydration gradient is approximately -2.0 wt%/km near the downdip in the southwestern part of the tectonic tremor-occurring area, whereas it is approximately -1.0 wt%/km near the centre of the tectonic tremor-generating area in the northeastern part (Fig. S8(b)). We calculated the vertical sum of the dehydration gradient from the slab surface to the slab Moho and found that its spatial pattern is oblique to the tectonic tremor-occurring area

and that the values are larger in the southwestern part of the tectonic tremor-generating area (Fig. S9). It is generally believed that the dehydrated water is brought to the plate interface and causes tectonic tremors, so it is difficult to explain the relationship between the two if their spatial distributions are oblique.

Therefore, we considered changing the slab geometry, which affects the thermal structure. In general, isodepth contours of the slab surface are approximately parallel to the trench axis, but as mentioned above, isodepth contours are oriented almost perpendicular to the trench axis in the northeastern part of the model (Fig. S1(a)). The hypocentre locations are used to determine the slab geometry of Slab2. In the northeastern part of the model, the number of earthquakes is small, so the reliability of the slab geometry may not be high (Fig. S1(b)). First, we created a slab geometry (Fig. S1(c)) by modifying the slab geometry of Iwamoto et al. (2022)[S10] (Fig. S1(a)) so that the isodepth contours in the red dashed box can be almost parallel to the trench axis. The new slab geometry was then created by taking the weighted average of 8:2, 5:5, and 2:8 for the depth distributions of Fig. S1(a) and Fig. S1(c). Among these three slab geometries, we selected the most suitable one, for which the weighted RMS of the residuals between the observed and calculated heat flow was the smallest and the region with a large dehydration gradient was not oblique to the tectonic tremor occurring area. As a result, the geometry with a 2:8 weighted average satisfied these conditions (Fig. S1(d)). In the Results and Discussion section of the main text, we showed the results of numerical simulations using this slab geometry.

Table S1 Convergence rates and directions of the oceanic plate with respect to the North American plate.[S7]

Period (Myr)	Convergence rate (cm/yr) and direction
0-3	4.33 (N37.1°W)
3-6	4.44 (N34.1°W)
6-9	4.67 (N20.0°W)
9-12	4.66 (N19.5°W)
12-15	4.95 (N19.9°W)
15-18 (Present)	4.69 (N21.5°W)

Table S2 Weighted RMS of residuals between observed and calculated heat flow

**RMS** (mW/m^2)		Effective friction coefficient			
		0.008500	0.01275	0.01700	0.02125
Slab geometry	Fig. S1(a)	8.89	8.72	9.58	11.2
	Fig. S1(c)	9.99	9.82	10.6	12.1
	Fig. S1(d)	9.65	9.49	10.3	11.9

Table S3 Thickness of each layer of the Pacific plate and Yakutat terrane

Thickness	Pacific plate	Yakutat terrane
Marine sedimentary layer	2 km	6 km[S11]
Oceanic crust	5 km	25 km[S11]

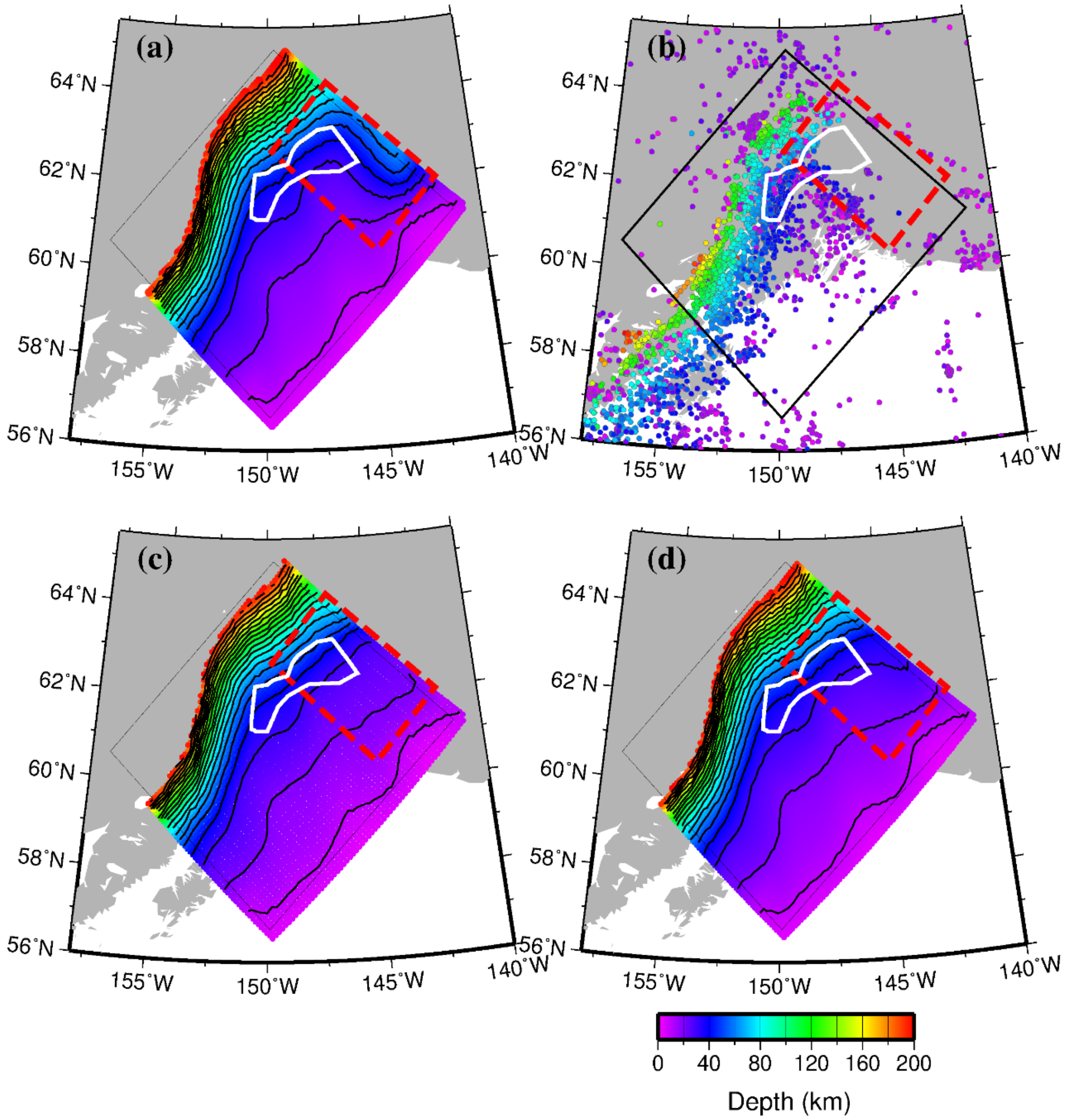

Figure S1 (a) Depth distribution of the slab surface by Iwamoto et al. (2022) [S10]. The thin black box represents the model region. The depth distribution is plotted only in the region where the depth of the slab surface is shallower than the bottom of the model (200 km), with a contour interval of 10 km. The white line indicates the area where tectonic tremors occur, as shown in Fig. 1. The area in the red dashed box is a region where the geometry of the slab surface was changed because of a small number of hypocentres. (b) Hypocentre distribution with local magnitudes of 3.0-4.0 occurring between January 1, 2010, and December 31, 2021[S12]. Hypocentres with a depth range of 0-200 km whose depth error, which is defined as the largest projection of the three principal errors on a vertical line, is less than ±2 km, are plotted. The thick black box represents the model region. The others are the same as in (a). (c) Same as (a) except that the depth distribution is modified from Iwamoto et al. (2022)[S10] by changing the slab geometry within the red dashed box so that the isodepth contours can be almost parallel to the trench axis. (d) Slab surface geometry created by weighted averages of (a) and (c) with a ratio of 2:8. Same as (a) except for the depth distribution of the slab surface used in this study. The map was created by using the Generic Mapping Tools (GMT)[S13] (version: GMT 4.5.7, URL link: https:// www. generic-mapping-tools.org/download/)

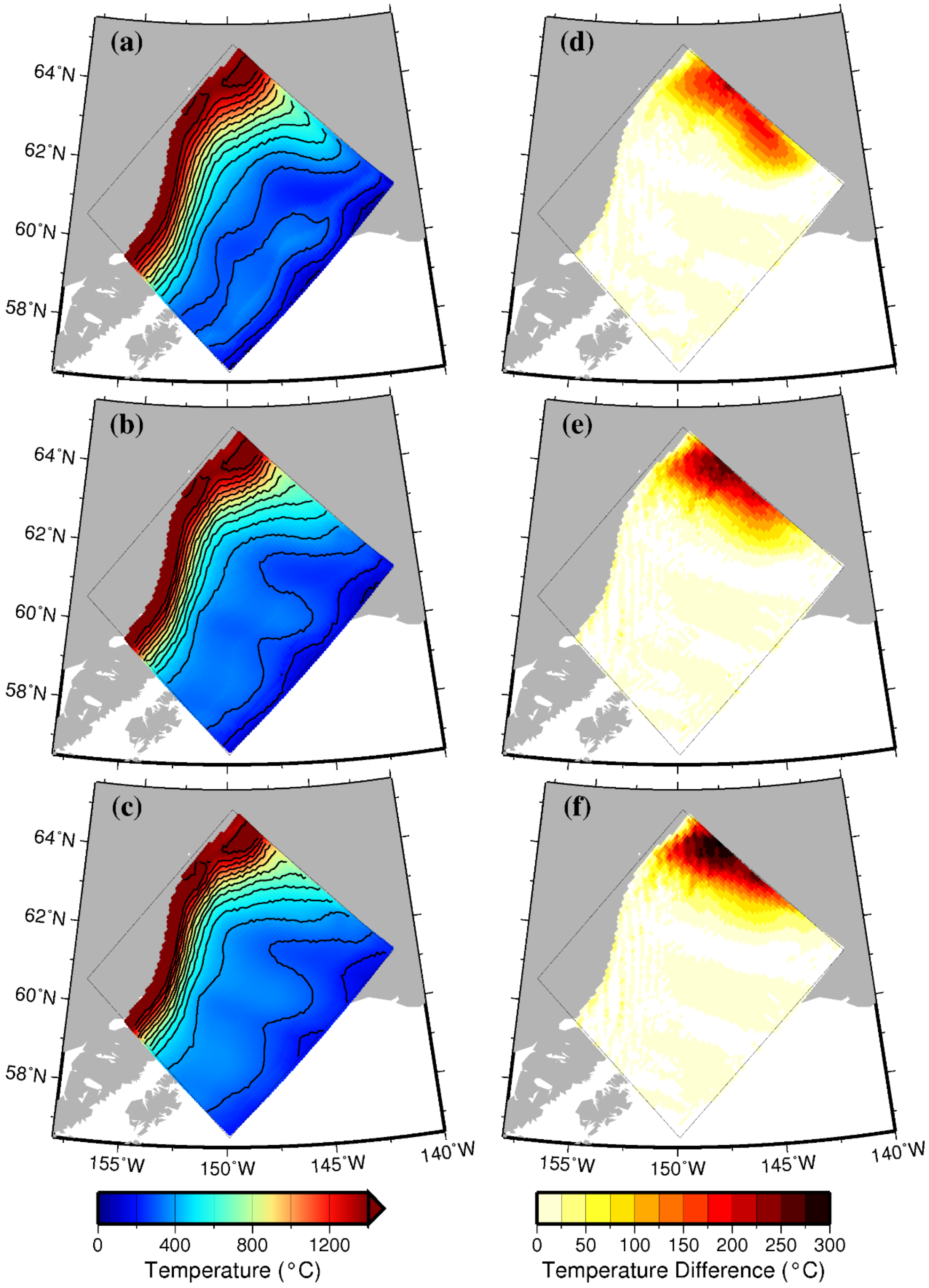
(a)
(b)
(c)
(d)
(e)
(f)
64°N
62°N
60°N
58°N
155°W
150°W
145°W
140°W
0
400
800
1200
Temperature (°C)
0
50
100
150
200
250
300
Temperature Difference (°C)

Figure S2 (a)-(c)Temperature distribution of the slab at present (0 Ma) obtained from Iwamoto et al. (2022)[S10]. The effective friction coefficient is 0.01275. The thin black box represents the model region. The temperature distribution is plotted only in the region where the depth of the slab surface is shallower than the bottom of the model (200 km), with a contour interval of 100 °C. (a) The slab surface (0 km). (b) 6 km depth from the slab surface. (c) 10 km depth from the slab surface. (d)-(f) Distribution of the temperature difference of the slab obtained from this study subtracted from that of Iwamoto et al. (2022)[S10]. (d) The slab surface (0 km). (e) 6 km depth from the slab surface. (f) 10 km depth from the slab surface. The map was created by using the Generic Mapping Tools (GMT)[S13] (version: GMT 4.5.7, URL link: https:// www. generic-mapping-tools.org/download/).

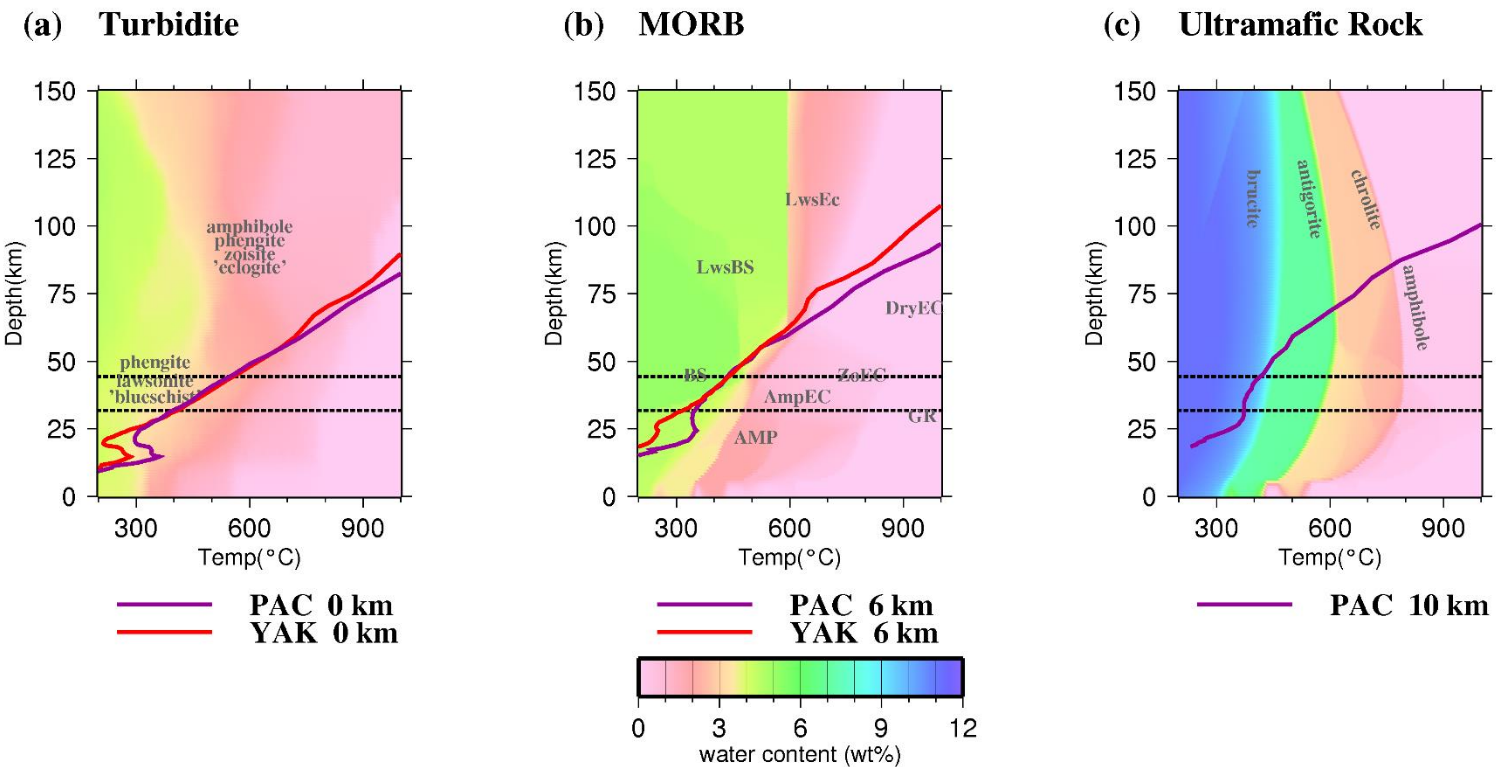

Figure S3 p-T paths and the depth range of tectonic tremors plotted on the phase diagrams. The horizontal black dotted line indicates the depth range (±1σ) of the tectonic tremors at the plate boundary. (a) Phase diagram of turbidite[S14]. The purple solid line shows the p-T path along the slab surface (0 km) of the subducted Pacific plate at Y = 200 km, and the red solid line shows the p-T path along the slab surface (0 km) of the subducted Yakutat terrane at Y = -200 km. (b) Phase diagram of MORB[S15]. . The purple solid line indicates a p-T path at a depth of 6 km from the slab surface of the subducted Pacific plate at Y = 200 km, and the red solid line indicates a p-T path at a depth of 6 km from the slab surface of the subducted Yakutat terrane at Y = -200 km. (c) Phase diagram of ultramafic rock[S15]. The purple solid line indicates a p-T path at a depth of 10 km from the slab surface of the subducted Pacific plate at Y = 200 km. The following acronyms are used: PAC, Pacific plate; YAK, Yakutat terrane; BS, blueschist; LwsBS, lawsonite blueschist; AMP, amphibolite; GR, granulite; AmpEC, amphibole eclogite; ZoEC, zoisite eclogite; LwsEC, lawsonite eclogite; and DryEC, dry eclogite.

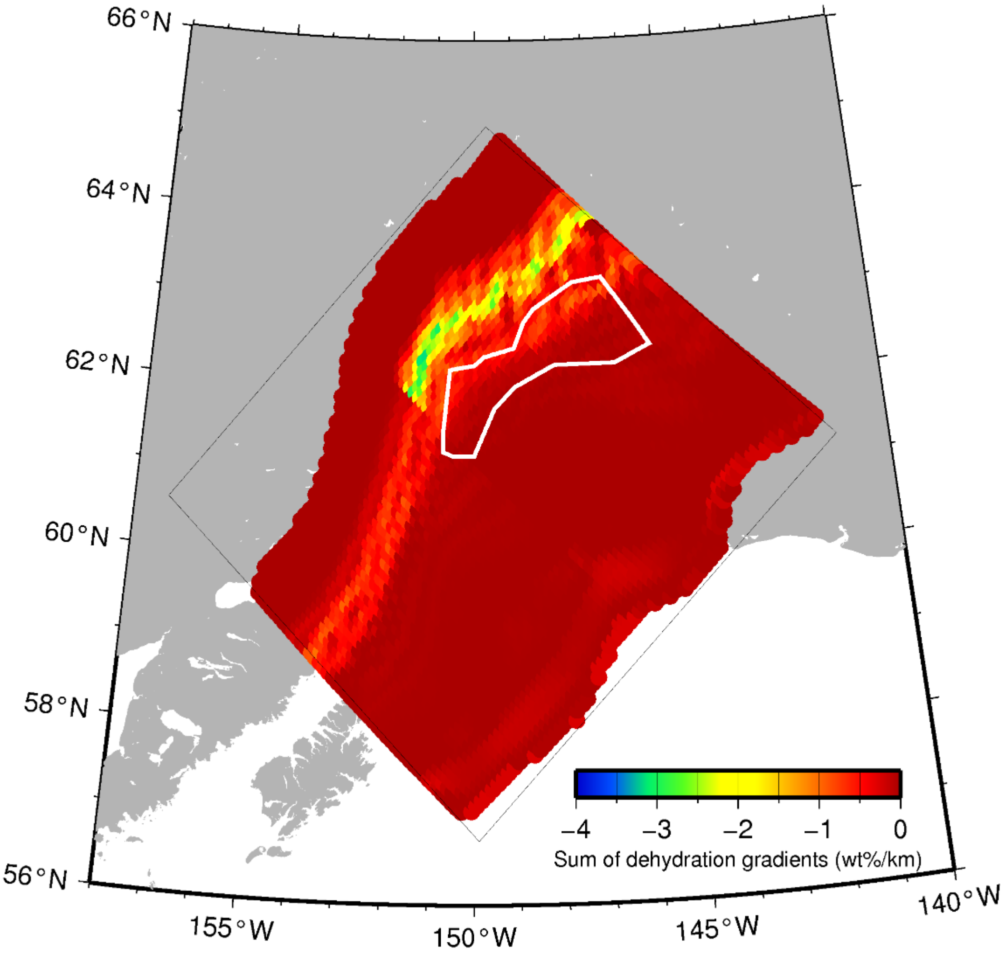

Figure S4 The distribution of the sum of dehydration gradients when the thickness of the sedimentary layer in the Yakutat terrane is assumed to be 2 km. The white line indicates the area where tectonic tremors occur, as shown in Fig. 1. It should be noted that this model used the same slab geometry model as Fig. S1(d). The others are the same as Fig. S2. The map was created by using the Generic Mapping Tools (GMT)[S13] (version: GMT 4.5.7, URL link: https:// www. generic-mapping-tools.org/download/).

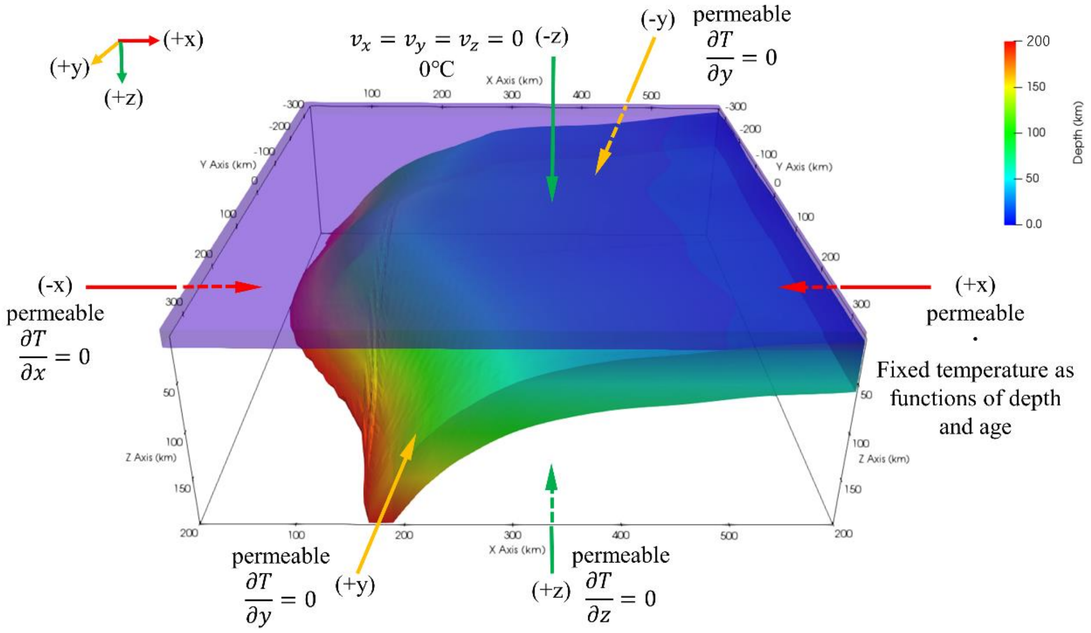

Figure S5 Boundary conditions of the model. The purple region at the top of the model represents the upper crust of the thermal conduction layer (Z = 0-16 km). The colour of the current (0 Ma) slab area indicates depth. The +x axis is taken from the inland side towards the trench axis, the +y axis is taken from the Yakutat terrane towards the Pacific plate along the trench axis, and the +z axis is taken in the depth direction. The red, yellow, and green arrows represent the ±x-, ±y-, and ±z-axis directions, respectively.

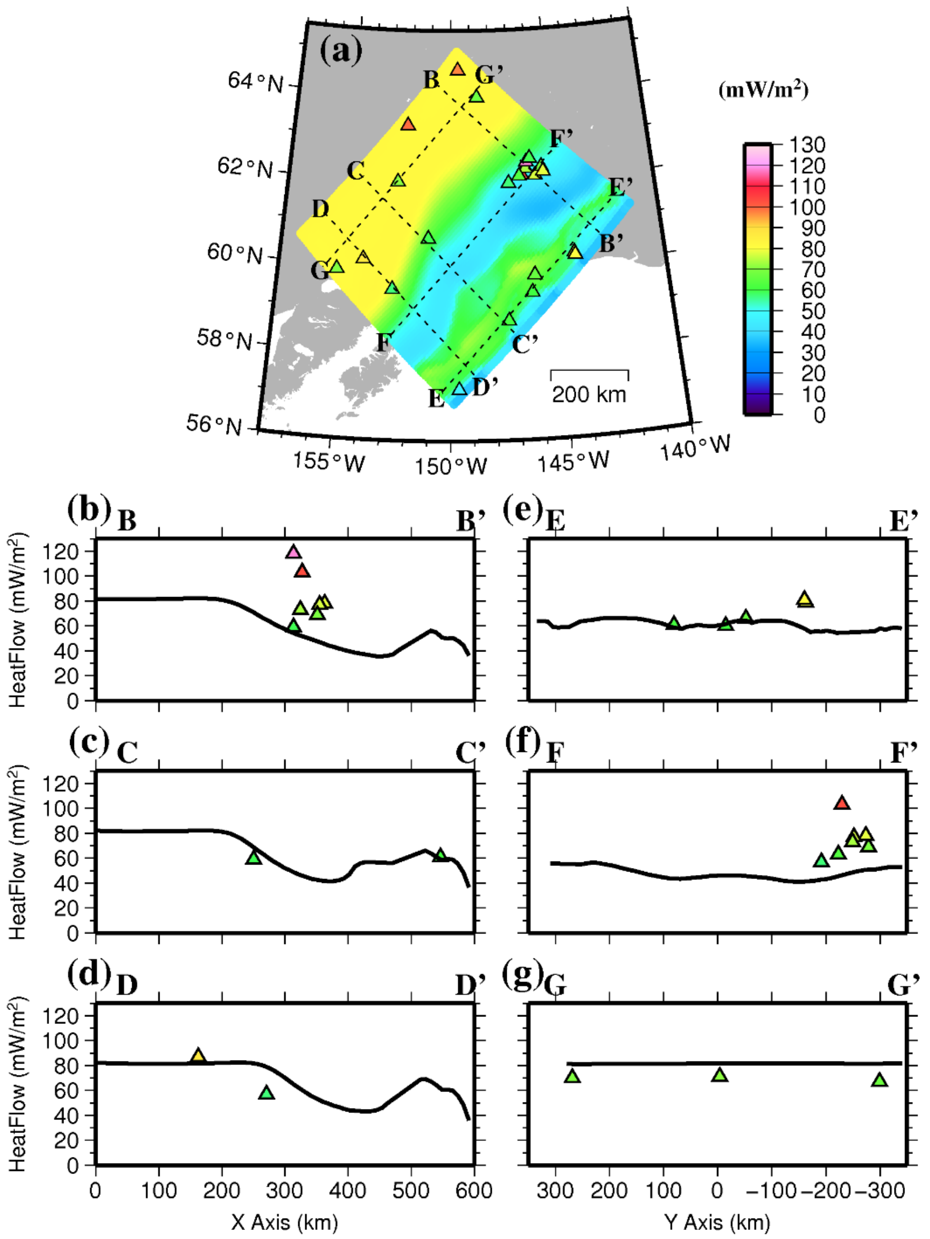

Figure S6 (a) Comparison between the observed [S8,S9] and calculated heat flow. The triangles are observation points coloured by the value of the observed heat flow. The colour contours show the spatial distribution of the calculated heat flow. (b) Comparison of heat flow along profile B-B' in (a). The black line denotes the calculation. The coloured triangles are observed heat flow within a one-sided width of 30 km. (c) Same as (b) except for profile C-C'. (d) Same as (b) except for profile D-D'. (e) Same as (b) except for profile E-E'. (f) Same as (b) except for profile F-F'. (g) Same as (b) except for profile G-G'. The map was created by using the Generic Mapping Tools (GMT)[S13] (version: GMT 4.5.7, URL link: https:// www. generic-mapping-tools.org/download/).

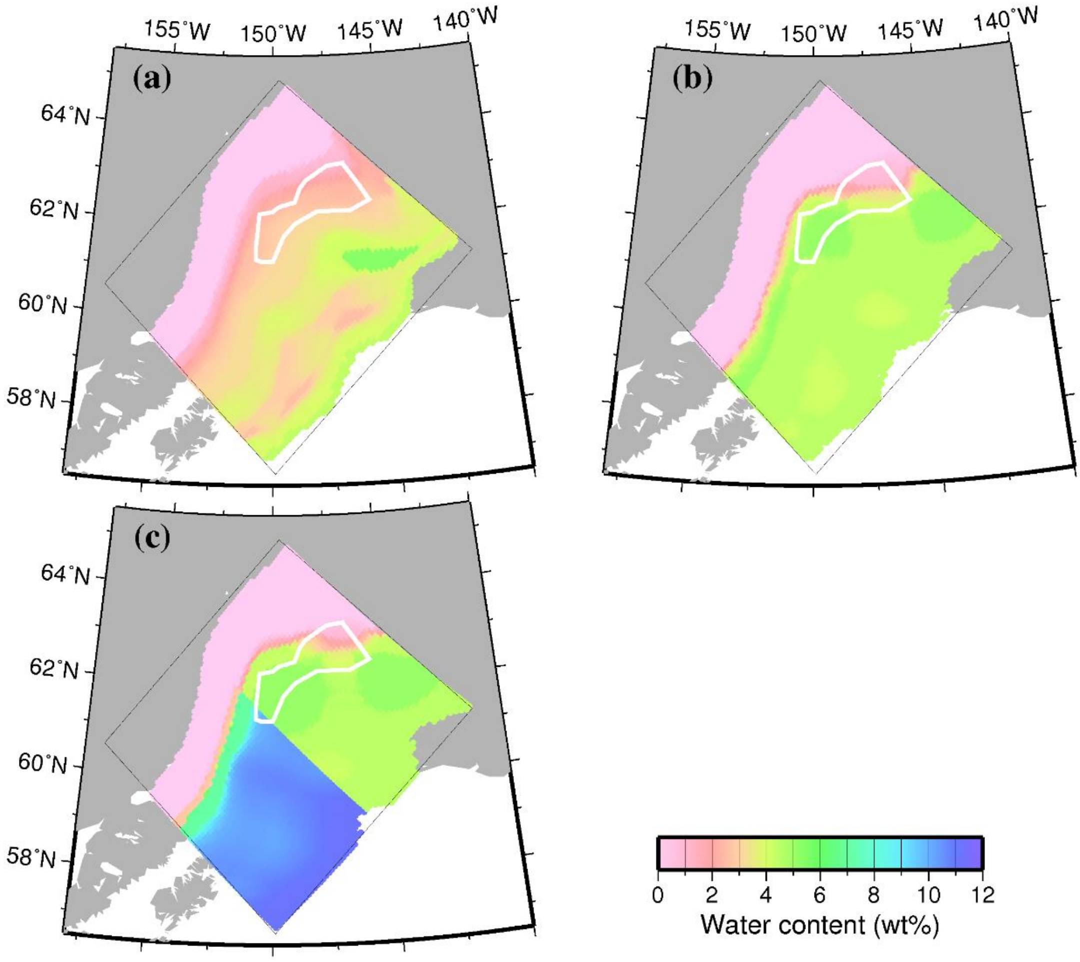

Figure S7 Same as Fig. S2 except that the water content distribution in the slab at present (0 Ma), which was calculated from the temperature distribution by Iwamoto et al. (2022)[S10], is plotted. (a) The slab surface (0 km). (b) 6 km depth from the slab surface. (c) 10 km depth from the slab surface. The map was created by using the Generic Mapping Tools (GMT)[S13] (version: GMT 4.5.7, URL link: https:// www. generic-mapping-tools.org/download/)

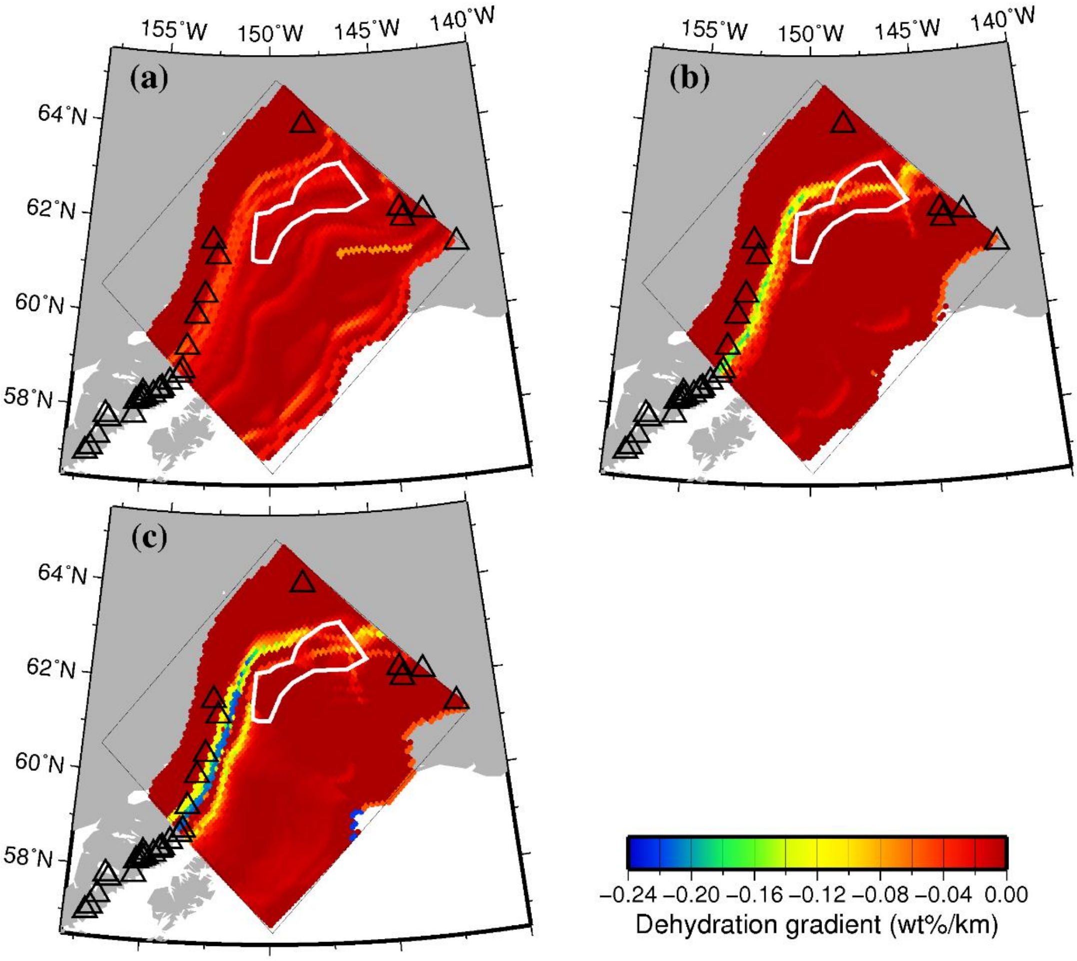

Figure S8 The dehydration gradient distribution in the slab at present (0 Ma), which was calculated from the temperature distribution by Iwamoto et al. (2022)[S10]. The open triangles represent volcanoes. The others are the same as Fig. S7. (a) The slab surface (0 km). (b) 6 km depth from the slab surface. (c) 10 km depth from the slab surface. The map was created by using the Generic Mapping Tools (GMT)[S13] (version: GMT 4.5.7, URL link: https:// www. generic-mapping-tools.org/download/).

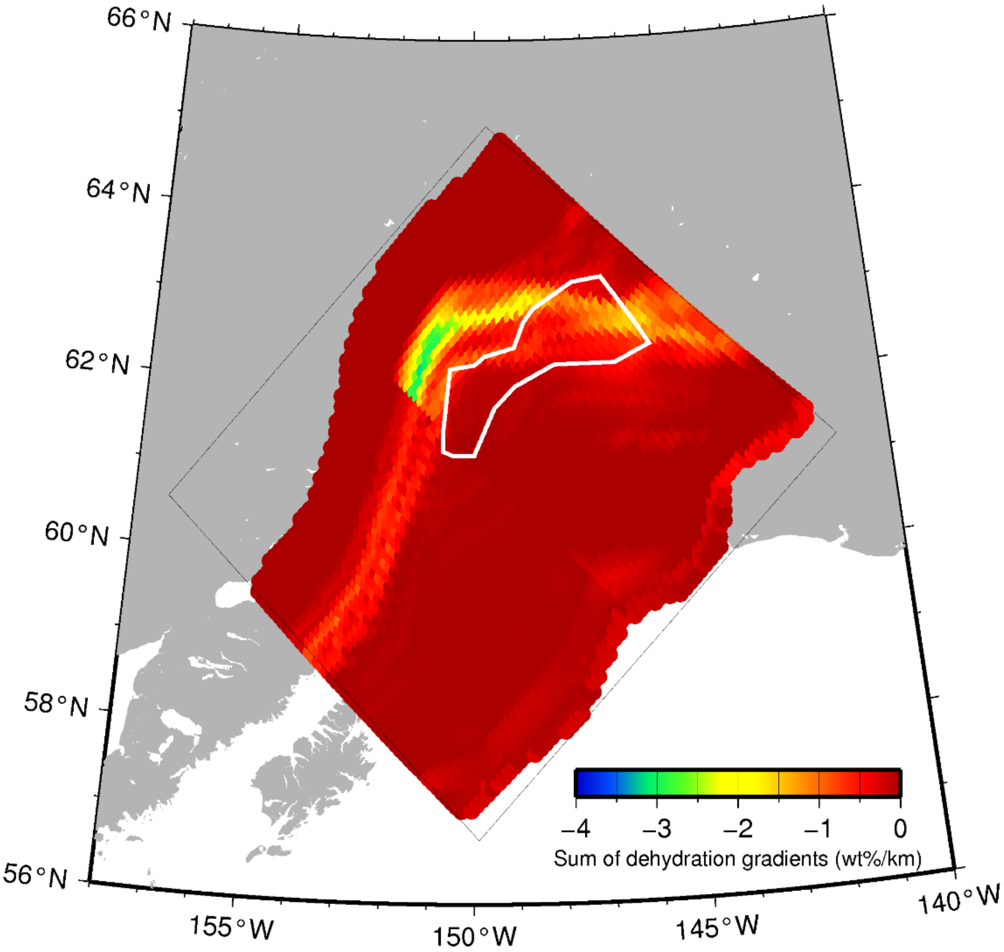

Figure S9 Same as Fig. S8 except for the vertical sum of the dehydration gradient distributions in the slab at present (0 Ma). The map was created by using the Generic Mapping Tools (GMT)[S13] (version: GMT 4.5.7, URL link: https:// www. generic-mapping-tools.org/download/).

References

S1. Yoshioka, S. & Sanshadokoro, H. Numerical simulations of deformation and dynamics of horizontally lying slabs. *Geophysical Journal International* **151**, 69-82 (2002).

S2. McKenzie, D. P. Some remarks on heat flow and gravity anomalies. *Journal of Geophysical Research* **72**, 6261-6273 (1967).

S3. Hayes, G. P. et al. Slab2, a comprehensive subduction zone geometry model. *Science* **362**, 58-61 (2018).

S4. Li, J. et al. Connections between subducted sediment, pore-fluid pressure, and earthquake behavior along the Alaska megathrust. *Geology* **46**, 299-302 (2018).

S5. Yoshii, T. Regionality of group velocities of Rayleigh waves in the Pacific and thickening of the plate. *Earth and Planetary Science Letters* **25**, 305-312 (1975).

S6. Ji, Y. & Yoshioka, S. Effects of slab geometry and obliquity on the interplate thermal regime associated with the subduction of three-dimensionally curved oceanic plates. *Geoscience Frontiers* **6**, 61-78 (2015).

S7. Matthews, K. J. et al. Global plate boundary evolution and kinematics since the late Paleozoic. *Global and Planetary Change* **146**, 226-250 (2016).

S8. Batir, J. F., Blackwell, D. D. & Richards, M. C. Updated Heat Flow of Alaska. *Alaska Energy Authority/Alaska Center for Energy and Power*, 47 (2013).

S9. University of North Dakota. *Global Heart Flow Database* https://engineering.und.edu/research/global-heat-flow-database/data.html

S10. Iwamoto, K., Suenaga, N., & Yoshioka, S. Temperature distribution for interplate seismic events in the south-central Alaska subduction zone based on 3-D thermal modeling. *Tectonophysics* (2022) (under review).

S11. Christeson, G. L. et al. The Yakutat terrane: Dramatic change in crustal thickness across the Transition fault, Alaska. *Geology* **38**, 895-898 (2010).

S12. United States Geological Survey. *Search Earthquake Catalog* https://earthquake.usgs.gov/earthquakes/search/

S13. Wessel, P. & Smith, W. H. New, improved version of Generic Mapping Tools released. Eos, Transactions American Geophysical Union 79, 579-579 (1998).

S14. van Keken, P. E., Hacker, B. R., Syracuse, E. M. & Abers, G. A. Subduction factory: 4. Depth-dependent flux of H2O from subducting slabs worldwide. *Journal of Geophysical Research: Solid Earth* **116** (2011).

S15. Tatsumi, Y., Suenaga, N., Yoshioka, S., Kaneko, K. & Matsumoto, T. Contrasting volcano spacing along SW Japan arc caused by difference in age of subducting lithosphere. *Scientific reports* **10**, 1-11 (2020).
